# Reversible Martensitic Transformation under Low Magnetic Fields in Magnetic Shape Memory Alloys

**DOI:** 10.1038/srep40434

**Published:** 2017-01-16

**Authors:** N. M. Bruno, S. Wang, I. Karaman, Y. I. Chumlyakov

**Affiliations:** 1 Department of Materials Science and Engineering, Texas A&M University, College Station, TX, USA; 2 Department of Mechanical Engineering, Texas A&M University, College Station, TX, USA; 3 Siberian Physical Technical Institute, Tomsk State University, Tomsk 634050, Russia

## Abstract

Magnetic field-induced, reversible martensitic transformations in NiCoMnIn meta-magnetic shape memory alloys were studied under constant and varying mechanical loads to understand the role of coupled magneto-mechanical loading on the transformation characteristics and the magnetic field levels required for reversible phase transformations. The samples with two distinct microstructures were tested along the [001] austenite crystallographic direction using a custom designed magneto-thermo-mechanical characterization device while carefully controlling their thermodynamic states through isothermal constant stress and stress-varying magnetic field ramping. Measurements revealed that these meta-magnetic shape memory alloys were capable of generating entropy changes of 14 J kg^−1^ K^−1^ or 22 J kg ^−1^ K^−1^, and corresponding magnetocaloric cooling with reversible shape changes as high as 5.6% under only 1.3 T, or 3 T applied magnetic fields, respectively. Thus, we demonstrate that this alloy is suitable as an active component in near room temperature devices, such as magnetocaloric regenerators, and that the field levels generated by permanent magnets can be sufficient to completely transform the alloy between its martensitic and austenitic states if the loading sequence developed, herein, is employed.

Meta-magnetic shape memory alloys (MMSMAs) have recently received much attention due to their ability to transform magnetic Zeeman energy into mechanical work and/or heat flow^1^,^2^,^3^,^4^,^5^,^6^,^7^,^8^,^9^,^10^,^11^. In these materials, magnetic field drives martensitic transformations and, therefore, the latent heat of the structural transition generates the giant inverse magnetocaloric effect (MCE)^12^,^13^,^14^,^15^,^16^,^17^,^18^,^19^. The NiCoMnIn MMSMAs studied here have been reported to exhibit temperature changes as large as 6 K across martensite to austenite magnetic-field-induced transformations^7^ (MFITs), however, large magnetic fields, i.e. above 2 T, are often needed to complete the transformation. Ideally, MMSMAs should completely transform under fields below 2 T for practical magnetocaloric refrigeration cycles due to the current maximal magnetic remanence in permanent magnets. Most of the experimental work on MMSMAs, to date, has focused on reducing their transformation hysteresis under applied magnetic fields, and thus the required field levels, by tuning their microstructure^20^,^21^,^22^,^23^,^24^.

Alternatively, magnetic hysteresis can also be reduced by carefully controlling the MMSMAs’ thermodynamic state. This state depends on the magnitude of coupled applied intensive thermodynamic forces including mechanical stress, magnetic field, and temperature. Extensive thermodynamic properties, e.g. strain, magnetization, and entropy serve as an indication of how much martensitic transformation occurs from applying these forces. Stress-magnetization or field-strain measurements are often difficult to perform and are rarely reported in literature^25^,^26^,^27^,^28^,^29^,^30^,^31^,^32^, thus limiting the current understanding of the role of MMSMAs’ thermodynamic state under mixed loading conditions on martensitic transformation characteristics and the magnetic field requirements to complete the transformation. It has been hypothesized, however, that applying special stress-field loading sequences should provide a means to control the thermodynamic state of MMSMAs and reduce the magnetic field levels for complete martensitic transformation to the levels below the maximal 2 T^7^, thus making MMSMAs more practical for magnetic refrigeration applications. Until now, experimental evidences corroborating this hypothesis have not widely been published.

In our previous work^26^, a custom Magneto-Thermo-Mechanical Characterization (MaTMeCh) device was developed lending us the ability to carefully control the MMSMAs’ thermodynamic state and drive martensitic transformations under both mechanical stress and magnetic fields across a wide temperature window. During the transformation, uniaxial strain, stress, volume average magnetization, applied field, and sample temperature can be simultaneously measured. Here, this device was used to demonstrate the applicability of MMSMAs for near-room temperature magnetocaloric cooling with a process we call varying stress-field ramping (VS-FR). When this process was employed, the complete martensitic transformation was triggered under magnetic fields below the desired 2 T limit. The data presented, here, includes the simultaneously measured applied field, volume average magnetization, uniaxial stress, strain, and temperatures across the multiferroic transitions in NiCoMnIn MMSMA single crystals oriented along the [001] austenite direction.

## Experimental Details

### Materials Fabrication and Processing

Ni_45_Co_5_Mn_36.6_In_13.4_ (nom. at.%) MMSMA samples were fabricated by vacuum induction melting of high purity constituents. Single crystals were then grown via the Bridgman method under He environment. The composition of the single crystals was measured using wavelength dispersive spectroscopy (WDS) at multiple points in the microstructure. The measured composition was close to the nominal and was found to be Ni_44.8_Co_5.0_Mn_36.0_In_14.1_ at. %. Single crystal compression samples with dimensions of 4 mm × 4 mm × 8 mm were cut with wire electro-discharge machining so that the longitudinal direction of the compression specimen corresponded to the [001] austenite crystal direction.

NiCoMnIn single crystals exhibit drastically different martensitic transformation characteristics when annealed to promote different degrees of long-range crystallographic order. Varying the degree of long-range order in these materials can be used to tune their magnetocaloric operating temperatures, i.e. the martensitic transformation temperatures and characteristics^33^,^34^,^35^,^36^. In turn, these characteristics influence the magnetic field levels needed to completely transform the alloy, which has been shown to influence the degree of achievable magnetocaloric cooling^37^,^38^,^39^,^40^,^41^. In the present study, we look at two cases of long range ordering and how they influence the required magnetic field to achieve a complete field-driven martensitic transformation.

Here, the single crystal samples were solution heat treated (SHT) at 1173 K for 24 hours and then quenched in water (WQ). Our intent was to grow a B2 order-dominant microstructure^34^,^36^. Secondary annealing below the reported L2_1_ to B2 ordering temperature (900 K)^35^ was also performed on some single crystal compression samples. The intent of these secondary annealing treatments was to change the long range ordering from B2 to L2_1_. The selected secondary heat treatments were performed at 873 K for 30 min^42^ followed by WQ after the samples were solution heat treated. This annealing time and temperature have been shown to sufficiently promote L2_1_ ordering in a number of previous works^21^,^34^.

During the solution and secondary annealing procedures, single crystals were wrapped in tantalum foil and sealed in quartz vials. During sealing, the vials were evacuated to vacuum (<1 · 10^−5^ Torr) at least 3 times and then vented with ultrahigh purity argon gas. The final argon pressure in the vial during the annealing treatment was 5 Torr.

### Experimental Protocols

Dark field imaging was performed to characterize the long-range order in annealed single crystals using a FEI Technai G^2^ field-emission transmission electron microscope. Since the austenite phase of these materials show concurrent B2 and L2_1_ crystallographic ordering^21^,^33^,^34^, dark field transmission electron microscopy (TEM) offers a means to visualize the relative amounts of these phases. TEM samples were prepared with twin jet polishing using a 1:3 nitric acid to methanol electrolyte under 20 V at 243 K. The images of L2_1_ morphology within the single crystals were collected along the [011] austenite zone axis using the (111) reflection peaks. Samples exhibiting different degrees of ordering, discussed above, were studied to determine which (B2 or L2_1_) ordering had the ability to minimize the magnetic field requirement needed for complete martensitic transformation.

A superconducting quantum interference device vibrating sample magnetometer (SQUID-VSM) was employed to measure the thermo-magnetic responses of the samples. These samples were first heated to 325 K under zero magnetic field and then 0.01 T was then applied along the [001] austenite direction. While the magnetization was measured, the samples were subsequently cooled at 5 K min^−1^ to 10 K followed by reheating to 325 K.

**Figure 1:**
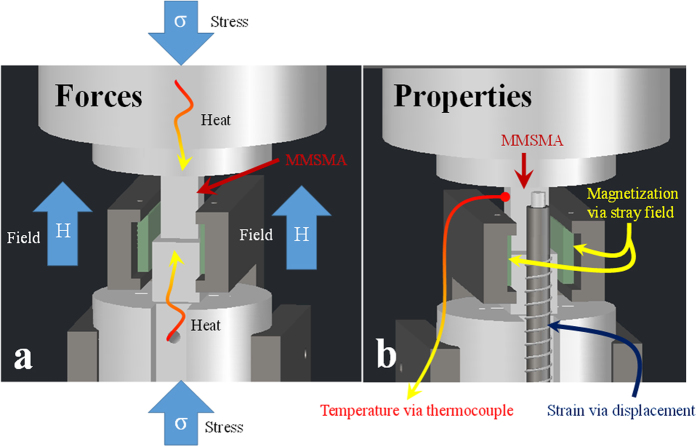
An illustration of the forces applied to the NiMnCoIn meta-magnetic shape memory alloy specimen within the multi-field magneto-thermo-mechanical characterization (MaTMeCh) device (**a**) and the corresponding materials properties simultaneously measured (**b**) during the mixed loading shown in (**a**).

The multi-field thermodynamic state of the MMSMA single crystals was controlled using the MaTMeCh device shown in Fig. 1 26. This device compressed the single crystal NiMnCoIn MMSMAs along the [001] austenite direction with drive rods connected to a custom actuator. The specimen and MaTMeCh device were inserted into a wide-bore superconducting magnet. The applied magnetic field generated in the magnet bore was, therefore, collinear to the applied stress as illustrated in Fig. 1a. The crystal orientation along the applied magnetic field in the MaTMeCh device matched the crystal orientation along the applied field in the SQUID-VSM. Various sensors and hardware including a capacitive displacement sensor, magnetic Hall sensors, and thermocouples within the instrument surrounded the single crystal and measured compressive stress-strain, stray-magnetic flux, and temperature, respectively, during the magneto-thermo-mechanical loading. The stray magnetic flux was used to compute the volume average magnetization level of the single crystal as described in ref. 26. The single crystals were mechanically compressed via displacement control at a strain rate of 2.5 · 10^−5^ s^−1^ and the magnetic field was ramped no faster than 50 Oe s^−1^. These values are generally accepted as slow enough to ensure the sample does not change temperature during isothermal measurements. This was verified with a thermocouple attached to the sample.

## Materials Characterization Results

### Magneto-microstructural Characterization

**Figure 2:**
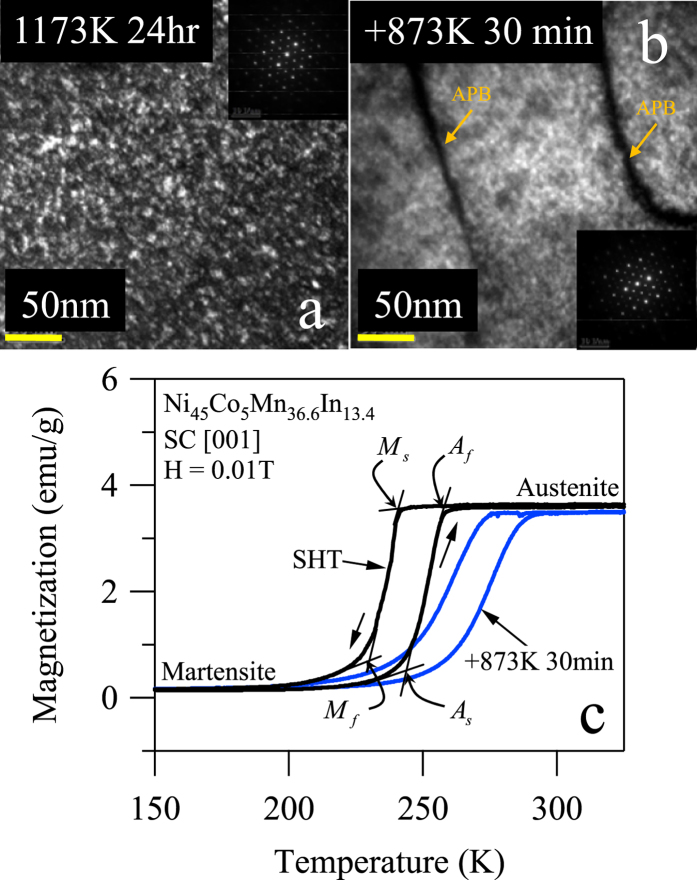
Dark field TEM micrographs of Ni_45_Co_5_Mn_36.6_In_13.4_ single crystals (SC) solution heat treated (SHT) at 1173 K for 24** **hrs (**a**) and secondary heat treated at 873 K for 30 min after SHT (**b**) showing the L2_1_ morphology and clear antiphase boundaries (APBs). The influence of secondary heat treatment on the thermo-magnetic response for each alloy is shown in (**c**) across the martensitic transition.

A dark field TEM micrograph depicting the long range B2 order in the SHT alloy is shown in Fig. 2a. In the figure, dark microstructural regions correspond to B2 order and the bright regions to L2_1_ order. The micrograph shows that the L2_1_ regions that form on quenching from 1173 K are, on average, no larger than 30 nm.

Figure 2b is a dark field image illuminating the L2_1_ morphology of the SHT + 873 K 30 min sample. The L2_1_ morphology appears to coarsen as a result of the secondary heat treatment. L2_1_ domains larger than 50 nm were observed separated by dark (B2 ordered) antiphase boundaries (APBs) labeled in the figure^43^,^44^,^45^,^46^. After secondary annealing, transformation temperatures had increased, as shown by the thermomagnetic responses in Fig. 2c.

The thermomagnetic data shown in Fig. 2c were used to approximate the stress/field-free martensitic transformation temperatures of both MMSMA sample conditions under 0.01 T (negligible field). The martensitic transformation temperatures, martensite finish (*M*_*f*_), martensite start (*M*_*s*_), austenite start (*A*_*s*_), and austenite finish (*A*_*f*_), of the SHT sample are denoted in the figure and were found to be 223 K, 242 K, 234 K, 261 K, respectively. Those of the SHT + 873 K 30 min sample were determined from Fig. 2c to be 225 K, 275 K, 248 K, and 291 K, respectively. Clearly, the heat treatment and change in long range order increased some of the martensitic transformation temperatures in the single crystals by nearly 25 K and decreased the thermal hysteresis, defined here as *A*_*f*_ − *M*_*s*_^47^, from 19 K to 16 K. As mentioned above, these transformation characteristics should influence the magnetic field levels needed to complete the martensitic transformation.

In Fig. 2c, the magnetization levels of the high temperature austenite phases were found to be nearly 3.5 emu g^−1^ under 0.01 T and the magnetization level of the martensite phases vanishes across the austenite to martensite transformation. On heating, the MMSMA recovers its magnetization on transforming back to austenite.

### Magneto-Thermo-Mechanical Characterization

**Figure 3:**
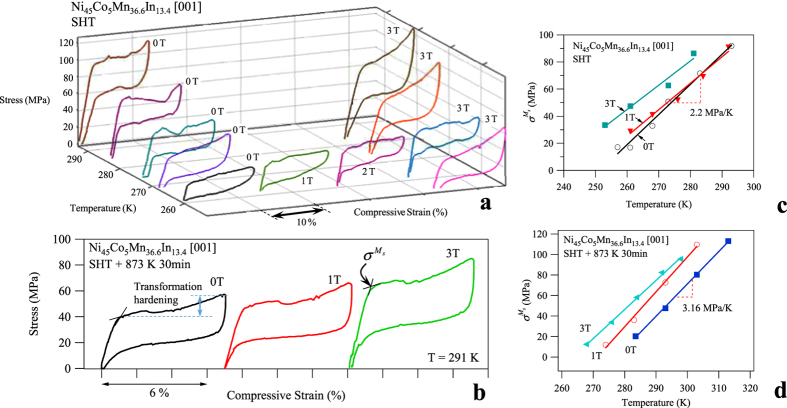
Superelastic responses of the solution heat treated (SHT) Ni_45_Co_5_Mn_36.6_In_13.4_ single crystals along the [001] austenite crystal direction between 250 K and 300 K, and between 0 T to 3 T magnetic field levels (**a**), the superelastic responses of SHT + 873 K 30 min Ni_45_Co_5_Mn_36.6_In_13.4_ single crystals at 291 K between 0 T and 3 T (**b**). The extracted phase diagrams from the critical martensite nucleation stresses, 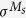 in (**a**) and (**b**) are plotted in (**c**) and (**d**), respectively.

The MaTMeCh device was used as described in the experimental details to characterize the MMSMA behaviors under multi-field loading and to extract thermodynamic quantities relevant to understanding their caloric behaviors and thermodynamic state. In Fig. 3a, the isothermal-isofield stress-strain curves are plotted over a wide field-temperature-stress window for the solution heat treated (SHT) NiCoMnIn single crystals. In Fig. 3a, the material was first tested at a temperature 2 degrees above the reported *A*_*f*_ temperature in Fig. 2c and under zero magnetic field (see 0 T curve at 263 K in Fig. 3a). On increasing the temperature of the compression sample under zero magnetic field, the stress level required to initiate and complete the stress-induced martensitic transformation also increased. Moreover, when larger field levels were applied to the single crystal at 263 K, a similar increase in the required stress levels was observed. This phenomenon is known as magneto-stress^1^ and is a result of the decrease in the free energy of the austenite phase, i.e. austenite is expected to become more thermodynamically favorable under larger fields.

In Fig. 3b, isothermal-isofield stress-strain curves were plotted for the SHT + 873 K 30 min annealed single crystal at 291 K, i.e. the *A*_*f*_ temperature. On the 3 T stress-strain curve, the critical stress indicating the onset of martensite nucleation is labeled as 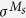. These critical stresses for the stress-strain curves in Fig. 3a and b were plotted against temperature and field for the SHT and SHT + 873 K 30 min annealed single crystals in Fig. 3c and d, respectively. Interestingly, the 1 T and 3 T curves in these plots are nearly parallel in both annealing cases suggesting that 3 T applied field has negligible effect on their slope. The slope of these lines is referred to as the Clausius-Clapeyron slope, or 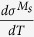, and is a thermodynamic quantity that can be used for computing the entropy change across a first order martensitic transformation. This entropy change is that responsible for the giant inverse magnetocaloric effect in MMSMAs. In the SHT and SHT + 873 K 30 min alloys, the Clausius-Clapeyron slopes were found to be 2.2 MPa K^−1^ and 3.16 MPa K^−1^, respectively.

In Fig. 3a and b, the transformation strain of the MMSMAs across the stress-induced transition (i.e. the strain at the point of 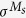 to the strain at the end of the mechanical loading) were nearly 6% for both annealing treatments. Therefore, we deduce that long range crystal ordering does not largely influence transformation strain in these alloys. Later, equilibrium thermodynamics will be used to compute the entropy change across martensitic transformations driven under constant stress or constant field conditions. The entropy change can be computed using both the Clausius-Clapeyron slope from Fig. 3c and d and the transformation strain measured under multi-field loading.

## Using Multi-Field Loading to Minimize Magnetic Field Requirements for Martensitic Transformation

A series of magneto-thermo-mechanical experiments were performed on the MMSMA specimens described above to determine if a complete martensite to austenite transformation could be driven using magnetic fields below 2 T. The full martensite to austenite transformation is needed to unlock the entire magnetocaloric cooling potential of MMSMAs. In practical magnetocaloric cooling applications, permanent magnets are typically used due to their small size and low cost^48^, but the magnitude of their magnetic remanence limits their field generation to nearly 2 T. This is normally insufficient to complete the stress-free martensite to austenite transformation in NiCoMnIn MMSMAs^49^. Therefore, constant mechanical stress–field ramping (CS-FR) and varying stress-field ramping (VS-FR) was performed on MMSMAs to induce the complete martensite to austenite to martensite transformation cycles and to determine if mechanical loading can, in fact, decrease the required magnetic field levels.

**Figure 4:**
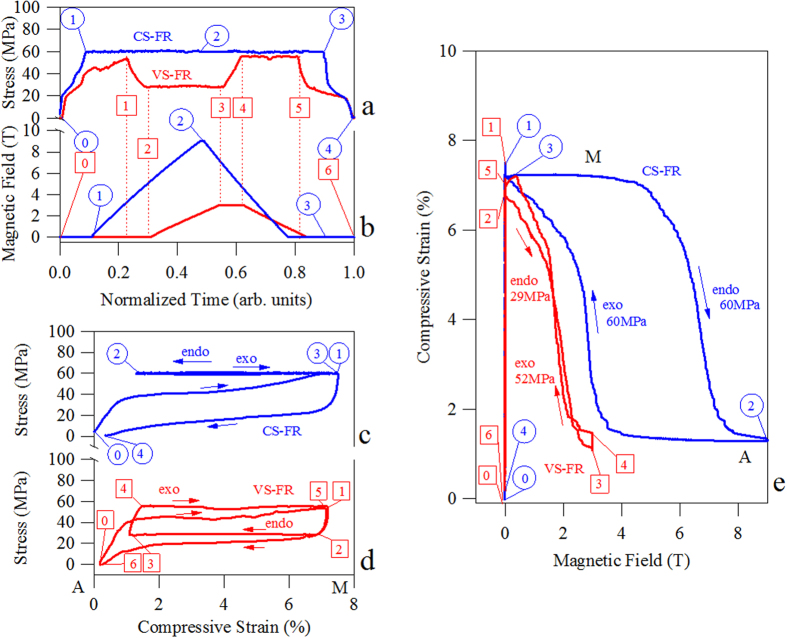
The stress-time (**a**) and magnetic field-time (**b**) loading sequence for the constant stress magnetic field ramping (CS-FR) and the varying stress magnetic field ramping (VS-FR) in Ni_45_Co_5_Mn_36.6_In_13.4_ single crystals after SHT + 873 K 30 min followed by water quenching (WQ). The stress-strain measurements from CS-FR (**c**) and VS-FR (**d**) loading sequences and the compressive strain- magnetic field measurements from CS-FR and VS-FR (**e**) loading processes were simultaneously measured. Circles indicate critical process points for CS-FR loading and boxes indicate critical process points in VS-FR loading. The steps are described in the text.

In Fig. 4a and b, the simultaneously applied stress and magnetic field ramping on the SHT + 873 K 30 min MMSMA specimen are shown for complete CS-FR (blue) and VS-FR (red) loading cycles, respectively. The loading sequence for each case is described in detail, below. The corresponding stress-strain responses are shown in Fig. 4c (CS-FR) and d (VS-FR).

In the figure, circled numbers correspond to starting points of each loading step in CS-FR loading and boxed numbers correspond to those in VS-FR loading. Both VS-FR and CS-FR loading cases begin by setting the MMSMA sample temperature to a temperature equal to or above its *A*_*f*_ temperature. In the case of Fig. 4, the sample was set to 291 K, i.e. the *A*_*f*_ shown in Fig. 1c. The austenite phase in the mixed loading experiments is always used as the reference starting phase.

### Constant Mechanical Stress – Field ramping Loading of Secondary Annealed Ni_45_Co_5_Mn_36.6_In_13.4_

In CS-FR, the MMSMA is initially compressed, quasi-statically, from point 0 to 1 with displacement-rate controlled compression (Fig. 4a). The stress-induced martensitic transformation from austenite to martensite is shown from point 0 to 1 in Fig. 4c and e. Between these two points, the magnetic field (see Fig. 4e) is held constant at 0 T. Point 1, here, corresponds approximately to the end of the stress-induced austenite to martensite transformation at nearly 6% transformation strain; this is near the reported maximum transformation strain along the [001] direction in this material^1^ as depicted in Fig. 3b. Next, the stress was held constant at this strain level, i.e. at 60 MPa, thus, the MMSMA is in a stress-stabilized martensitic state at 291 K. Then, the magnetic field was applied collinear-to-stress up to 9 T as indicated by points 1 to 2. Figure 4c and e show the simultaneously measured strain data across the CS-FR (blue) loading. It is important to note that the strain generated from applying 9 T is equal to 5.86% indicating the martensite to austenite reverse transformation occurred and the endothermic reaction responsible for the giant inverse MCE was generated. During the loading sequence, the sample temperature remained constant due to sufficiently slow magnetic field ramping and temperature control, mentioned above.

After reaching point 2 in the CS-FR loading sequence the magnetic field was unloaded from point 2 to 3 and the stress was held constant. Unloading the field resulted in the austenite to martensite transformation as shown by the return strain path in Fig. 4c and e. After the magnetic field was completely removed, the stress was then removed (see points 3 to 4 in Fig. 4a and b), from the stress-stabilized martensite, and the stress-stabilized martensite transformed back to stress-free austenite at 291 K, as shown in Fig. 4c. A small amount of irrecoverable strain (between points 0 and 4) measured can be attributed to remnant martensite near *A*_*f*_ temperature.

Loading the MMSMA with the CS-FR process resulted in a substantial magnetic hysteresis across the martensite to austenite transition. Figure 4e shows that the martensitic transformation from the stress-stabilized martensite to austenite at 291 K did not start until nearly 5 T was reached and the transformation did not complete until above 8 T. The CS-FR was characterized by 4 T magnetic hysteresis and, as can be seen in the figure, very large magnetic fields were needed to generate the martensitic transformation under a constant mechanical load.

### Varying Stress-Field Ramping Loading of Secondary Annealed Ni_45_Co_5_Mn_36.6_In_13.4_

Similarly, the VS-FR loading process was plotted in red at 291 K for the sample with the same annealing treatment. The process steps are labeled with boxed numbers in Fig. 4. Initially, the sample was mechanically pre-loaded from austenite to martensite so subsequent field ramping could induce the martensite to austenite transformation. In this particular sample, the complete field-free austenite to martensite transformation is achieved with 52 MPa (point 0 to 1) uniaxial stress as indicated by the strain level matching that of the other sample. As shown in Fig. 4c and d, a slightly lower uniaxial stress was needed to reach the same level of transformation strain in the VS-FR sample. Next, the uniaxial stress was unloaded to 29 MPa (point 1 to 2), which corresponded to the onset of the field free martensite to austenite transformation. This mechanical “preloading” and “unloading” was performed under zero magnetic field as shown in Fig. 4b. At point 2 in Fig. 4d, it can be seen that the sample was on the verge of transforming from stress-stabilized martensite to austenite. The strain values are also plotted in Fig. 4e for different points. At point 2, the magnetic field was then ramped until the full transformation strain was measured (points 2 to 3 in Fig. 4b and e) while the stress was held constant. As shown in Fig. 4e, 5.59% strain was achieved from magnetic field ramping up to 3 T. This is nearly equivalent to the CS-FR experiment in terms of the attained strain level. The endothermic reaction associated with the martensite to austenite transformation can be achieved with significantly less magnetic field using the VS-FR loading rather than the CS-FR sequence.

Once 3T was applied in the VS-FR loading path, the compressive stress was increased back up to 52 MPa under a constant 3 T (points 3 to 4) rather than immediately unloading the field. The small elastic strain associated with this mechanical loading can also be seen in Fig. 4e at 3 T. At point 4, the stress was held constant at 52 MPa while the magnetic field was removed (points 4 to 5 in Fig. 4a and b). This resulted in the austenite to martensite transformation and the associated exothermic reaction. The sample transformed to its initial thermodynamic state, i.e. “point 1”, after removing the magnetic field. Once the magnetic field was removed, the sample was mechanically unloaded (point 5 to 6) and the martensite to austenite transformation occurred since the temperature was maintained at 291 K.

The VS-FR loading depicted in Fig. 4c resulted in nearly 30 MPa stress hysteresis. In the case of CS-FR loading (points 1–2–3 in Fig. 4c), the stress hysteresis is zero, but energy was still dissipated across the martensitic transformation as indicated by the magnetic hysteresis in Fig. 4e. Conversely, magnetic hysteresis is minimized by the VS-FR sequence. In Fig. 4d and e, the VS-FR loading process resulted in 5.59% strain, which was nearly equal to that of the CS-FR loading process (5.86%), thus the exothermic and endothermic reactions from the martensitic transformation are expected to be nearly equivalent no matter the loading path.

The Clausius-Clapeyron (CC) relation, 
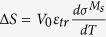
, was employed to quantify the entropy change across CS-FR and VS-FR loading. In Fig. 3d, the CC slope, 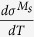, of the secondary annealed alloy was shown to be 3.16 MPa K^−1^. Approximating the MMSMA’s specific volume, *V*_0_, as 1.25 · 10^−4^ m^3^ kg^−1^, the entropy change for CS-FR and VS-FR loading was computed to be 23 J kg^−1^ K^−1^ and 22 J kg^−1^ K^−1^, respectively. The benefit of the VS-FR process instead of the CS-FR, however, is the minimization of the magnetic hysteresis, and thus, the lower required magnetic field levels to achieve a nearly complete martensitic transformation. VS-FR loading minimizes the magnetic hysteresis and the MMSMA will completely transform under 3 T when the magnetic field is applied and removed under different stress levels.

### Effect of Microstructure on Transformation Temperatures

It has been reported that the magnetic field levels needed to complete the martensitic transition are linked to the martensitic transformation range, i.e. *M*_*s*_ − *M*_*f*_^16^. In turn, the transformation range has been shown to influence the so-called “transformation hardening”^50^,^51^. This hardening is defined by the difference in the martensite nucleation stress, 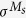, and the stress level needed to complete the austenite to martensite transformation at a constant temperature. Both 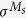 and transformation hardening are illustrated in Fig. 3b. Large transition ranges and transformation hardening are controlled by the MMSMA microstructure, such as long range order, dislocations, and second phases. Thermodynamically speaking, large transformation hardening is an indication that the magnetic fields needed to complete the martensitic transformation must overcome large microstructural barriers. As shown in Fig. 3b on the 0 T stress-strain curve, the transformation hardening of the SHT + 873 K 30 min sample was nearly 27 MPa. This significant hardening also manifests as a large transition range (*M*_*s*_ − *M*_*f*_) shown in the thermo-magnetization curve in Fig. 2c for the SHT + 873 K 30 min sample. As shown in Fig. 2c, the transition range of the SHT + 873 K 30 min aged sample was 50 K, however, the transition range of the SHT sample was nearly 22 K. Comparing the microstructures in Fig. 2a and b, we believe that antiphase boundaries (APB’s) must play a role in the microstructural propagation of martensite during transformation. For instance, the SHT sample shown in Fig. 2a does not exhibit any clear APBs. The SHT + 873 K 30 min sample, on the other hand, shows clear L2_1_ morphology separated by large APBs. Perhaps, the local lattice strain energy around APBs acts as pinning sites for martensite propagation which could explain the larger transition range and transformation hardening in the SHT + 873 K 30 min annealed samples. Since the SHT microstructure, show in Fig. 2a did not have any clear APBs, it was expected that a SHT sample would exhibit smaller transformation hardening and require magnetic fields that were below 3 T to complete the transformation.

### Varying Stress-Field Ramping Loading of Solution Heat Treated Ni_45_Co_5_Mn_36.6_In_13.4_

**Figure 5:**
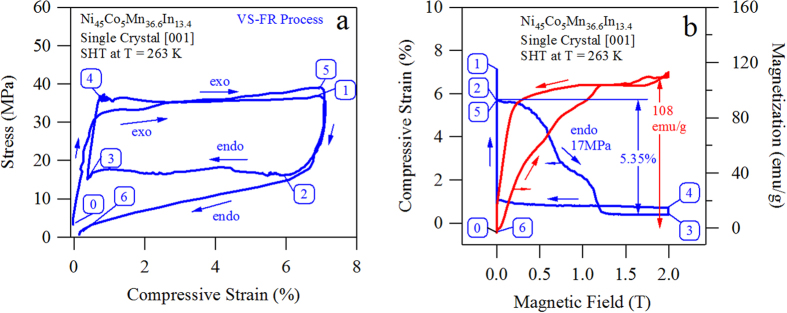
The stress-strain measurements for VS-FR loading sequence in solution heat treated (SHT) water quenched (WQ) Ni_45_Co_5_Mn_36.6_In_13.4_ single crystals. Critical process points for VS-FR loading are indicated and correspond to the loading sequence depicted by the red curve in Fig. 4a and b. The steps are described in the text. The simultaneously measured strain and magnetization measurements (**b**) for VS-FR loading are also plotted indicating the complete martensitic transformation can be driven with magnetic fields below 2 T.

The SHT sample was subjected to VS-FR loading two degrees above its *A*_*f*_ temperature, i.e. 263 K. The simultaneously measured stress-strain and strain-magnetization-field results are plotted in Fig. 5a and b, respectively. Thermodynamic state of the MMSMA was numbered corresponding to the VS-FR loading sequence in Fig. 4a and b, however the stress and field levels that were applied to the SHT alloy were different than those on the SHT + 873 K 30 min sample due to their microstructural differences.

Initially, the SHT compression sample was mechanically loaded in austenite to a strain level equal to the transformation strain (nearly 6%); this corresponded to a mechanical load of 36 MPa as shown from point 0 to 1. Next, the load was decreased to 17 MPa, or the onset of the martensite to austenite transition, resulting in a mechanically preloaded sample. While holding the load constant at 17 MPa, 2 T field was applied collinear to the load. This resulted in a magnetic field induced strain and a magnetization increase from points 2 to 3 as shown in Fig. 5a and b. Interestingly, the MMSMA completes the martensite to austenite transformation under only 1.3 T as indicated by the 5.35% magnetic field induced strain and 100 emu g^−1^ magnetization increase in Fig. 5b. To the authors’ knowledge, this is the first time this material has been shown to exhibit the complete magnetic field-induced (and stress-assisted) martensite to austenite transition with magnetic field levels below 2 T.

From point 2 to 3 in Fig. 5, the MMSMA exhibits the endothermic reaction responsible for the giant inverse MCE. At point 3, the magnetic field is then held constant and the stress is increased to point 4. At point 4, the stress is held constant and the magnetic field is removed. In the SHT sample, the austenite to martensite transformation did not initiate until the magnetic field was completely removed, thus a small magnetic hysteresis is present in Fig. 5b. After the field was removed, the martensitic transformation occurred from point 4 to 5 as a result of the applied stress. This suggests that the nucleation of martensite was more difficult in the presence of magnetic field.

The CC slope of the SHT NiCoMnIn single crystal was measured and shown in Fig. 3c to be 2.2 MPa K^−1^. Per the Clausius-Clapeyron equation mentioned above, VS-FR loading generated an entropy change of approximately 14.7 J kg^−1^ K^−1^. This entropy change is smaller than the secondary heat treated alloy, the reasons of which will be discussed in a future works and related to the microstructural differences shown in Fig. 2a and b and the magnetic ordering in the samples. However, the complete transformation was driven with a magnetic field of only 1.3 T, which makes the access to this entropy change practical with permanent magnets.

Achieving the complete transformation under a magnetic field of only 1.3 T can be attributed to the small transformation hardening (and *M*_*s*_ − *M*_*f*_ transition temperature range) of only 7 MPa in the SHT sample. The L2_1_ morphology in Fig. 1a and b suggest that the smaller L2_1_ morphology observed in the B2 ordered SHT case results in an easier propagation of the stress-induced martensite, thus lower transformation hardening. In the SHT + 873 K 30 min case, the L2_1_ regions are very large and are separated by clear B2-L2_1_ anti-phase boundaries. Currently the exact correlation between the martensite crystal structure dependence on austenite ordering is not fully understood, but known that it changes the crystallographic modulation of the martensite. Therefore, more work is needed to link transformation hardening to crystallographic order and martensite crystal structure and variant size scales.

## Conclusions

In conclusion, the complete martensite to austenite transformation was achieved with only 1.3 T in Ni_45_Co_5_Mn_36.6_In_13.4_ (at.%) single crystals. The new magneto-mechanical loading process used to achieve the complete reversible transformation, namely, varying stress-field ramping (VS-FR) was employed. Here, we also demonstrated the MMSMA transformation response from typical constant stress-magnetic field ramping (CS-FR) to characterize martensitic transformations under magnetic field and mechanical load, and then investigated the influence of applying and removing the magnetic field under varying stress levels. Employing a varying stress state while field ramping (as opposed to constant stress) effectively reduces magnetic hysteresis in the Ni_45_Co_5_Mn_36.6_In_13.4_ (at.%) MMSMAs from 4 T to nearly zero, and the required magnetic field levels for a complete transformation from 8 T to 1.3 T. Therefore, the applicability of employing NiCoMnIn MMSMAs in household refrigeration processes with magnetic field levels equal to those attainable in permanent magnets has been demonstrated. It is possible to design a solid state refrigerator which can apply the VS-FR loading sequence, which will be topic of a follow up publication.

## Additional Information

**How to cite this article:** Bruno, N. M. *et al*. Reversible Martensitic Transformation under Low Magnetic Fields in Magnetic Shape Memory Alloys. *Sci. Rep.* **7**, 40434; doi: 10.1038/srep40434 (2017).

**Publisher's note:** Springer Nature remains neutral with regard to jurisdictional claims in published maps and institutional affiliations.
